# Repression of the *fliC* gene as an immune evasion strategy in *Yersinia ruckeri* infection of rainbow trout *(Oncorhynchus mykiss)*

**DOI:** 10.2478/jvetres-2025-0059

**Published:** 2025-10-22

**Authors:** Patrycja Schulz, Joanna Pajdak-Czaus, Karolina Pospiech, Elżbieta Fornal, Amanda Kobiera, Justyna Matczak, Paweł Foksiński, Andrzej Krzysztof Siwicki

**Affiliations:** Department of of Ichthyopathology and Fish Health Prevention, National Inland Fisheries Research Institute, 10-719 Olsztyn, Poland; Department of Epizootiology, Faculty of Veterinary Medicine, University of Warmia and Mazury in Olsztyn, 10-719 Olsztyn, Poland Proteon Pharmaceuticals S.A., 90-364 Łódź, Poland; Proteon Pharmaceuticals S.A., 90-364 Łódź, Poland; Department of Microbiology and Clinical Immunology, Faculty of Veterinary Medicine, University of Warmia and Mazury in Olsztyn, Proteon Pharmaceuticals S.A., 10-719 Olsztyn, Poland Proteon Pharmaceuticals S.A., Proteon Pharmaceuticals S.A., 90-364 Łódź, Poland

**Keywords:** fish yersiniosis, gene silencing, immune evasion, virulence attenuation, virulence genes

## Abstract

**Introduction:**

The virulence of *Yersinia ruckeri*, the causative agent of enteric redmouth disease in salmonids, is influenced by multiple factors, including flagellar gene expression. This study investigates the role of *fliC* gene expression in the pathogenicity of *Y. ruckeri* and its impact on the immune response of infected rainbow trout (*Oncorhynchus mykiss*).

**Material and Methods:**

Using two virulent strains differing in *fliC* expression, clinical symptoms, mortality rates and key immune parameters were evaluated. Ninety farmed rainbow trout with average body weight of 110.5 ± 24.1 g and average length of 20.7 ± 1.9 cm were used. Allocation was made of 10 fish each to a control group, a low-dose group challenged with one strain, a high-dose group challenged with that strain, and low- and high-dose groups challenged with the second strain, and each challenge group was duplicated.

**Results:**

Fish infected with the *fliC*-repressed strain exhibited more severe symptoms, higher mortality rates and a weaker immune response regardless of infectious dose compared to those infected with the *fliC*-expressing strain. The lack of an active *fliC* gene was associated with a lower gammaglobulin level, decreased respiratory burst and suppressed T-cell proliferation. However, increased potential killing activity was noted for that strain.

**Conclusion:**

These findings clearly demonstrate the dual role of the *fliC* gene in the pathogenicity of *Y. ruckeri* and host immune modulation in rainbow trout.

## Introduction

Aquaculture is one of the most rapidly expanding sectors within the global food production industry, and it is scaling up to meet the increasing demand for its products. Besides providing a staple of human nutrition, fish also hold substantial economic importance (9, 32). Among the various species, rainbow trout (*Oncorhynchus mykiss*) is the most widely introduced and cultivated salmonid fish globally. The aquaculture production of rainbow trout occurs in South America, Europe, Asia, Africa and Oceania, and thus well beyond its native habitat in North America (9). Its widespread popularity can be attributed to several factors, including ease of breeding, rapid growth rates and adaptability to a variety of environments. However, the industry breeding it faces notable challenges, with diseases being the primary factor contributing to significant losses in fish stocks. Addressing these challenges is crucial for sustaining the growth and viability of aquaculture as a key component of global food security (23).

One of the most significant bacterial diseases affecting salmonid aquaculture is caused by *Yersinia ruckeri*, a Gram-negative enterobacterium with a global distribution. This pathogen is responsible for fish yersiniosis, commonly referred to as enteric redmouth disease (ERM), which manifests as a systemic infection (3). The disease leads to generalised septicaemia in the infected host, as the bacteria disseminate throughout the body *via* the bloodstream. This triggers an inflammatory response in various tissues, resulting in external haemorrhaging and the formation of small petechiae around and within the oral cavity, which is the origin of the disease’s name. Other clinical signs include exophthalmos, skin darkening, lethargy and anorexia. Pathological examinations reveal splenomegaly and inflammation of the posterior intestine, often characterised by an accumulation of thick yellow mucus. Petechiae may also be observed on the serosal surfaces and within muscle tissues. Petechial haemorrhages can also be evident on the liver, pancreas, pyloric caeca, swim bladder and muscles. Fluid accumulation may lead to abdominal distension (16). The disease typically initiates with the mortality of individual fish, ultimately resulting in elevated mortality rates across the entire population. In rainbow trout, mortality can reach 70%. The significance of *Y. ruckeri* is also increasing as a pathogen in Atlantic salmon (*Salmo salar*), where it causes a condition known as “salmonid blood spot,” which is generally less severe than ERM (3). Over the past few years, *Y. ruckeri* has resulted in fatal outbreaks in Nile tilapia (*Oreochromis niloticus*) (1) and channel catfish (*Ictalurus punctatus*) (30); therefore, it seems to be a threat to a broadening range of aquaculture species. During outbreaks, complete eradication of the pathogen from affected aquaculture facilities proves challenging, one reason for which is that *Y. ruckeri* can persist in bottom sediments for several months and form biofilms on tank walls. Infected fish may develop a carrier state and be pathogen reservoirs. It is estimated that up to 25% of fish on a farm may be carriers, which will shed the pathogen for up to two months post infection. This dynamic contributes to recurrent and cyclical disease occurrences on certain fish farms (22).

The severity of a disease is influenced by the specific strain of bacteria responsible for the infection. During the infection process, bacterial pathogens employ specific genes known as virulence factors (VFs) to interact with the host, which results in damage to host tissue or disease (17, 32). Recent advancements in alternative technologies, including proteomic, transcriptomic and genomic analyses, have facilitated the identification of potential VFs in bacteria such as *Yersinia ruckeri*. Many of the VFs identified thus far pertain to extracellular factors that are common among a broad range of Gram-negative pathogenic bacteria, particularly within the enterobacteria group. Key extracellular molecules involved in the infection process of *Y. ruckeri* include extracellular proteases, haemolysins and siderophores. Other notable VFs encompass outer membrane proteins, lipopolysaccharides, heat-sensitive factors, zinc and cysteine transporters and flagellar components (11, 32). The flagellum represents a complex surface structure that is essential for motility and serves various additional functions, including chemotaxis, biofilm formation and host attachment. It is primarily composed of monomeric flagellins, with FliC protein being recognised as critical in these processes and playing a vital role in bacterial pathogenesis in fish. Several genes are involved in flagella expression; however, the flagellin gene *fliC* encodes the subunits that make up the bulk of the flagellum’s structure. In the context of *Y. ruckeri*, flagellar proteins have been demonstrated to elicit a strong innate immune response, which can confer protection to fish. Furthermore, *Y. ruckeri* possesses a regulatory system that can sense its host and respond by suppressing the expression of *fliC* (15, 20). In this study, the effects of infection in rainbow trout were examined using two virulent strains of *Y. ruckeri* that differ in their expression of the *fliC* gene. The aim was to differentiate them by their impacts on clinical symptoms, mortality rates and humoral and cellular immune parameters and elucidate the effect of *fli*C repression as an immune evasion strategy.

## Material and Methods

### Bacterial strains

In this study, two *Y. ruckeri* isolates were used: strain 024PP2020, which has the *fliC* gene repressed; and strain R57, which expresses it. Both isolates were derived from fish in Polish ponds exhibiting symptoms of yersiniosis and are part of the laboratory strain collection of the National Inland Fisheries Research Institute.

### Culture conditions

Two independent methods were utilised to isolate the selected strains. The *Y. ruckeri* 024PP2020 strain grows on CHROMagar *Pseudomonas* medium (CHROMagar, Paris, France), forming small purple colonies. The *Y. ruckeri* R57 strain does not grow on this medium, so it was inoculated onto tryptic soy agar (TSA) medium (Biomaxima, Lublin, Poland). The samples were incubated for 48 h ± 2 h at 25°C ± 3°C. Subsequently, one typical purple colony from the CHROMagar *Pseudomonas* medium and four random colonies from the TSA medium were collected for genomic verification.

### Molecular and genomic analyses

Isolation of DNA was carried out from the bacterial suspension in 200 μL of phosphate-buffered saline (Pol-Aura, Dywity, Poland). The suspensions were thoroughly vortexed and DNA was isolated with the Wizard SV Genomic DNA Purification System (cat. No. A2361; Promega, Madison, WI, USA). A master mix was prepared for the lysis of bacterial cells by mixing 200 μL of Nuclei Lysis Solution, 50 μL of ethylenediaminetetraacetic acid 0.5 M at pH 8.0, 5 μL of RNase and 2 μL of proteinase K, which is not part of the system kit (20 mg/mL, activity ≥ 30 U/mg; A&A Biotechnology, Gdansk, Poland). DNA concentration was measured at the 260 nm wavelength and its purity was assessed by A260/230 and A260/280 ratios using a BioPhotometer D30 spectrophotometer (Eppendorf, Hamburg, Germany) and a 1-mm μCuvette G1.0 adaptor (Eppendorf).

A two-step PCR was performed for genomic verification. In the first step the *tuf* gene was targeted, which assigned the bacterial DNA to the *Yersinia* genus, and in the second step the *yrp, yer* and *yh1A* genes were amplified, which made finer assignment possible to the *Y. ruckeri* species (22). The reaction master mix in the first step consisted of 25 μL of Taq PCR Master Mix, 22 μL of PCR-grade water (cat. No. E2520; EURx, Gdańsk, Poland), 1 μL of each primer (Genomed, Warsaw, Poland) and 1 μL of DNA matrix. The master mix for the second step differed from that of the first in containing 18 μL of PCR-grade water and 3 μL of each primer. The PCR was performed using a nexus GX2 thermal cycler (Eppendorf). The reaction products were separated electrophoretically on 2% w/v agarose gel (cat. No. E0301; EURx). There was a 100 bp DNA marker (cat. No. SM0321; Thermo Scientific, Vilnius, Lithuania) on each gel. Visualisation of amplification products was performed using a UviTec device (UviTec, Cambridge, UK).

The 024PP2020 and R57 strains were subjected to paired-end 2× 150 base-pair next-generation sequencing on the Illumina platform. Data processing and preliminary analyses were performed using the Tormes 1.2.1 pipeline (25). Sequence types were determined by applying different multi-locus sequence typing (MLST) schemes. The schemes for *Yersinia* species take into account six or seven housekeeping genes – the McNally scheme designed for *Yersinia* species identification and diversification considers seven genes (*aarF, dfp, galR, glnS, hemA, rfaE* and *speA*) (12, 14), whereas the scheme described by Bastardo *et al*. (4) includes six (*dnaJ, glnA, gyrB, hsp60, recA* and *thrA*) and was designed specifically for *Y. ruckeri* species. However, because the differentiating resolution of MLST is low in the case of *Y. ruckeri*, additionally core-genome (cg) typing was performed by means of the cgMLSTFinder 1.1 tool (7, 35). Analysis of the presence of virulence genes was carried out using the VFanalyzer online platform based on the Virulence Factor DataBase (6, 19).

### Preparation of bacterial inoculum

The bacterial strains were stored in tryptic soy broth (TSB; Sigma-Aldrich, St. Louis, MO, USA) with 50% glycerol and maintained at -80°C until needed. The isolates were revived by plating them on TSA (Sigma-Aldrich) and incubating for 48 h at 25°C. The bacterial strains were transferred to TSB to create a 1% suspension and incubated with shaking at 140 rpm at 25°C until the suspensions reached an optical density (OD) of approximately 1.0 at 600 nm, corresponding to an estimated concentration of 1 × 10^8^ colony-forming units (CFU)/mL based on prior calibration for *Y. ruckeri* strains. The bacterial suspension was then centrifuged at 4,500 rpm for 20 min. After removal of the supernatant, the bacterial pellets were resuspended in 0.85% NaCl to obtain final concentrations of 1 × 10^7^ and 1 × 10^6^ CFU/mL. All suspensions were verified and, if necessary, adjusted using the plate count method to ensure accuracy.

### Experimental set-up

Ninety rainbow trout were obtained from a local fish farm and brought to the experimental facility at the Faculty of Veterinary Medicine, University of Warmia and Mazury in Olsztyn. The average body weight of the fish was 110.5 ± 24.1 g, and their average length was 20.7 ± 1.9 cm. The fish were kept in an experimental recirculating aquaculture system (RAS) equipped with temperature sensors and a UV lamp. The RAS consisted of fish tanks with a capacity of 180 L supplied with aerated tap water, one compensating tank with a capacity of 200 L and a pump tank with a capacity of 300 L. A programme providing 12 h of light and 12 h of darkness was implemented. During the experiment, the temperature, dissolved oxygen level and pH were monitored using SL 1000 equipment (Hach, Loveland, CO, USA), and they were maintained at the following levels: temperature at 16.7 ± 0.2°C; pH at 8.07 ± 0.10 and O2 level at 8.56 ± 0.47 mg/L. The fish were acclimatised to laboratory conditions for 7 d. Throughout the experiment, the fish were given a commercial feed recommended by the breeder at a rate of 1.5% of their body weight per day. The fish were randomly divided into control and experimental groups (Table 1). After the experimental infection, the recirculation of water was changed to flow-through so that the infection would not be transmitted between tanks.

**Table 1.** Experimental groups of rainbow trout challenged with *fliC* gene–repressed (024) and *fliC* gene–expressing (R57) *Yersinia ruckeri* strains

Group	Bacterial strain	Infectious dose (CFU/fish)	n	Repetitions
C	-	-	10	1
024-L	024PP2020	2 × 10^5^	10	2
024-H	024PP2020	2 × 10^6^	10	2
R57-L	R57	2 × 10^5^	10	2
R57-H	R57	2 × 10^6^	10	2

1 CFU – colony-forming units; C – control group; L – low infectious dose group; H – high infectious dose group

### Challenge test

For the challenge, the fish were anaesthetised using MS-222 (Sigma-Aldrich) and were intraperitoneally injected with 0.2 mL of *Y. ruckeri* resuspended in sterile 0.85% NaCl. The non-infected control group received an intraperitoneal injection of 0.2 mL of sterile 0.85% NaCl. The fish were monitored three times a day for 14 d, during which clinical symptoms, anatomical changes in internal organs and mortality were recorded. A swab from the kidney was collected from all dead or moribund fish and incubated on non-selective agar plates to re-isolate bacteria and confirm the cause of mortality. After 14 d the survivors were euthanised by an overdose of MS-222. Blood and organ samples were collected to determine non-specific immunity parameters.

### Sample collection and immune cell isolation

Blood was collected from the caudal vein and transferred to sterile tubes. Following centrifugation at 2,000 × *g* and 4°C for 10 min, serum was collected and stored at –20°C until the determination of humoral parameters was performed. The spleen and head kidney of each fish were removed aseptically and examined immediately after collection. Organs were pressed through a 60-μm nylon mesh. Organ immune cells were isolated using Gradisol L density-gradient centrifugation (Aqua-Med, Łódź, Poland). The isolated cells were suspended in Roswell Park Memorial Institute (RPMI)-1640 medium supplemented with 10% foetal calf serum and 1% antibiotic-antimycotic solution (both reagents from Sigma-Aldrich), dispensed into 96-well plates, incubated at 24°C and used for the assays. These evaluated the respiratory burst activity and the potential killing activity of spleen phagocytes and the proliferative response of head kidney lymphocytes.

### Serum humoral immune parameters

A turbidimetric assay was used to measure lysozyme activity in serum. The assay was based on the lysis of *Micrococcus lysodeikticus* bacteria (Sigma-Aldrich). A solution of the bacteria in a sodium phosphate buffer solution was mixed with serum. First immediately after the addition of bacteria and a second time after 1 h of incubation at 25°C, the absorbance was measured at 450 nm with a DR3900 spectrophotometer (Hach). Lysozyme activity was determined by subtracting the final from the initial absorbance.

Serum ceruloplasmin activity was determined using the spectrophotometric method. Serum was incubated for 15 min in an acetate buffer containing 0.2% p-phenylenediamine (Sigma-Aldrich). The reaction was stopped with sodium azide solution (Sigma-Aldrich). A Dynatech MRX 3 microplate reader (Dynex Technologies, Chantilly, VA, USA) was used to measure ceruloplasmin activity at 540 nm. The spectrophotometric method based on the principles of the biuret reaction, was used for the determination of total serum protein. Samples were incubated at room temperature for 30 min, after which their OD was determined at 540 nm with the Hach DR3900 spectrophotometer.

Gammaglobulin was precipitated from the serum with polyethylene glycol. The serum samples were mixed and incubated at room temperature for 2 h. After incubation, the samples were centrifuged and the supernatant was collected. The OD was determined at 540 nm with the Hach DR3900 spectrophotometer. By subtracting the OD values of the supernatant from those of the total protein, total serum Ig levels were calculated.

### Functional assays of immune cells

Phagocyte respiratory burst activity (RBA) was measured spectrophotometrically, based on the reduction of nitroblue tetrazolium salt (NBT). At the end of the incubation for 24 h at 22°C, non-adherent cells were removed. The medium was replaced with an NBT (nitroblue tetrazolium, Sigma-Aldrich) solution with or without phorbol myristate acetate (PMA; Sigma-Aldrich). The plates were incubated for 30 min at 24°C. Next, the NBT-containing medium was removed and the wells were rinsed with absolute and 70% ethanol. Blue formazan produced in cells was dissolved in 2 M KOH (Chempur, Piekary Śląskie, Poland) and dimethylsulfoxide (DMSO; Avantor Performance Materials (formerly POCh), Gliwice, Poland). Solution OD was measured at 620 nm in a Sunrise microplate reader (Tecan, Grödig, Austria). All samples were tested in triplicate and the results were expressed as the stimulation index (SI), which was calculated by dividing the mean OD of PMA-stimulated cells by the OD of the unstimulated control cells.

The potential killing activity (PKA) of splenic phagocytes was measured spectrophotometrically. After incubation and removal of the non-adherent cells, the cell suspension was mixed with NBT solution with or without *Aeromonas hydrophila* and incubated at 24°C for 30 min. The supernatant was removed from each well. Adherent cells were fixed with absolute ethanol and 70% ethanol. Then 2 M KOH and DMSO were added to each well. The Tecan Sunrise microplate reader was used to measure the amount of extracted reduced NBT at 620 nm. All samples were assayed in triplicate. Results were expressed as SI which was calculated by dividing the mean OD of bacteria-stimulated cells by the OD of the unstimulated control cells.

The mitogenic response of pronephros lymphocytes was determined using a 3-(4,5-dimethylthiazol-2-yl)-2,5-diphenyltetrazolium bromide (MTT) colorimetric assay (Sigma-Aldrich). Head kidney immune cells were cultured in the presence of mitogens: concanavalin A as a T cell mitogen, or lipopolysaccharide (LPS) from *Escherichia coli* as a B cell mitogen. Both mitogens were purchased from Sigma-Aldrich and used at concentrations of 10 μg cm^−3^) for 48 h at 22°C. A medium without mitogens was used to maintain unstimulated control cells. After incubation, MTT solution was added to each well and incubated at 24°C for 3 h. The supernatants were removed, DMSO was added to each well and the plates were incubated at room temperature for 15 min. After incubation, solubilised reduced MTT was measured colorimetrically at 570 nm with the Tecan microplate reader. All samples were tested in triplicate and results were reported as the SI, which was calculated by dividing the mean OD of the mitogen-stimulated cells by the OD of the unstimulated control cells.

### Data analysis

Mean values and standard deviations were used for comparisons between groups. All data are expressed as mean + SEM. After validation of normality with the Shapiro–Wilk test and homogeneity of variances with the Brown–Forsythe test, data were subjected to a two-sided *post-hoc* Dunnett’s multiple comparison test. If the assumption of normality was not met, the Kruskal–Wallis test and Dunn’s multiple comparison test were used. All calculations were determined to be significant at P-value ≤ 0.05. GraphPad Prism 10 software package (GraphPad Software, San Diego, CA, USA) was used for all statistical analyses.

## Results

### Molecular and genomic analyses

As determined by sequence typing (Table 2), *Y. ruckeri* 024PP2020 and R57 were found to have identical sequence types in both schemes. Both sequences were identified as ST44 in the McNally scheme, and both as ST33 in the Bastardo scheme. *Yersinia* species have 1,553 loci analysed in cgMLST typing, which assures much higher differentiation resolution. Despite this much higher number of investigated loci, the cgST sequence type also turned out to be the same for both strains and was cgST1927. Both strains also shared a cluster of 18 genes involved in the biosynthesis of legionaminic acid, which is a major component of the O-part of the LPS antigen in many Gram-negative bacteria, suggesting their common association with the O1 serotype.

**Table 2.** Multi-locus sequence typing (MLST) and core-genome MLST (cgMLST) for *Yersinia ruckeri* infection model strains

Isolate	Typing scheme	Sequence type	Housekeeping genes in the scheme (reference)						
*Y. ruckeri* 024PP2020 *Y. ruckeri* R57	MLST McNally (12)	44 44	*aarF* (13) *aarF* (13)	*dfp* (10) *dfp* (10)	*galR* (14) *galR* (14)	*glnS* (13) *glnS* (13)	*hemA* (15) *hemA* (15)	*rfaE* (14) *rfaE* (14)	*speA* (14) *speA* (14)
*Y ruckeri* 024PP2020 *Y. ruckeri* R57	MLST Bastardo (4)	33 33	*dnaJ* (1) *dnaJ* (1)	*glnA* (1) *glnA* (1)	*gyrB*(1) *gyrB*(1)	*hsp60* (1) *hsp60* (1)	*recA* (5) *recA* (5)	*thrA* (5) *thrA* (5)	- -
*Y ruckeri* 024PP2020 *Y ruckeri* R57	cgMLST	1927 1927				- -			

With the aim of potential biotype determination, 024PP2020 and R57 strains were analysed in terms of 34 genes associated with flagella activity and biosynthesis *(flgA, flhA, flhB, flhC, flhD, flhE, fliA, fliC, fliD, fliE, fliF, fliG, fliH, fliI, fliJ, fliN, fliO, fliP, fliR, fliS, fliT, fliZ, flgB, flgC, flgD, flgF, flgG, flgH, flgM, flgN, motA, DJ39_RS_06590, DJ39_RS_06615* and *DJ39_RS_06625)*. For strain 024PP2020 two mutations were observed within genes *flhC* and *fliF*, both resulting in a premature stop codon and presumably shorter and non-functional protein formation; the strain also only had a partial *fliC* sequence. For strain R57 a mutated *fliF* gene was identified. Since biological tests revealed motile biotypes for both strains, it is evident that the described mutations within flagella-related genes were not sufficient for mobility loss. Identification attempts found 91 VFs for the *Y. ruckeri* 024PP2020 strain and 92 for the R57 strain. Both strains differed from each other only by the presence of the *fliC* gene (Supplementary Table S1).

### Clinical manifestations

**Fig. 1.**
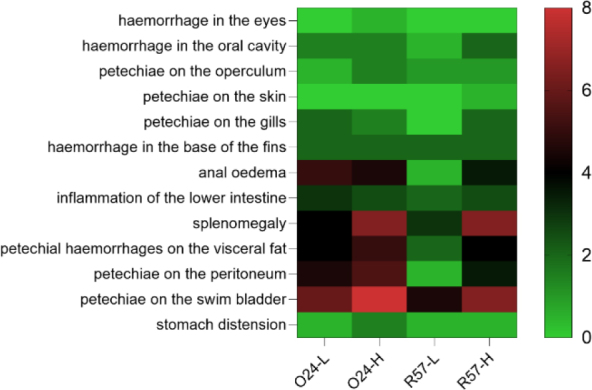
Frequency of clinical symptoms and pathological changes in rainbow trout challenged with *fliC* gene–repressed (024) and *fliC* gene–expressing (R57) *Yersinia ruckeri* strains. The result is the average of two repetitions. L – low infectious dose; H – high infectious dose. Colour gradient corresponds to the mean number of individuals showing clinical symptoms

All rainbow trout infected with *Y. ruckeri* exhibited characteristic clinical signs. These signs included haemorrhaging in the mouth and eyes, petechiae on the skin and gills, subcutaneous haemorrhages at the base of the fins, abdominal swelling and anal oedema with hyperaemia. Anatomopathological examination revealed visceral petechiae, particularly in the swim bladder, as well as splenomegaly, enteritis accompanied by catarrhal exudate and stomach distension. The severity of clinical signs and anatomical changes varied based on the strain and infectious dose. Notably, the 024-L group displayed more severe symptoms and anatomical changes than the R57-L group (Fig. 1).

### Mortality

**Fig. 2.**
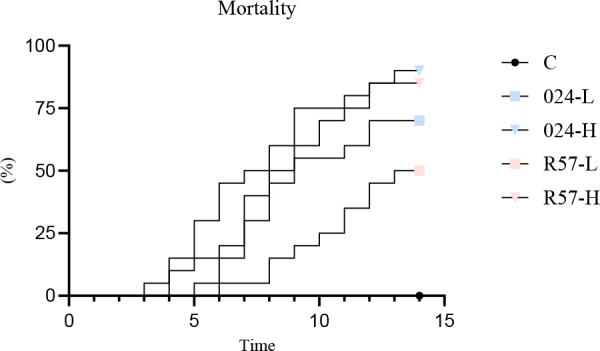
Mortality of rainbow trout challenged with *fliC* gene–repressed (024) and *fliC* gene–expressing (R57) *Yersinia ruckeri* strains. Mortality in the negative control group lies along the X-axis. L – low infectious dose; H – high infectious dose

All dead trout from the challenged groups were found to be positive for *Y. ruckeri* through pathogen re-isolation. Mortality occurred earlier in fish infected with 024PP2020 than in those infected with R57, regardless of the dose. Specifically, 024PP2020 resulted in mortality at 3 and 5 days post infection (dpi), while R57 did so at 4 and 6 dpi. When fish were infected with 024PP2020 at low and high doses, the mortality rates were 70% and 90%, respectively. In contrast, the same doses of the R57 strain led to lower mortality rates of 50% and 85%. Uninfected control fish displayed 100% survival (Fig. 2).

### Serum humoral immune parameters

**Fig. 3.**
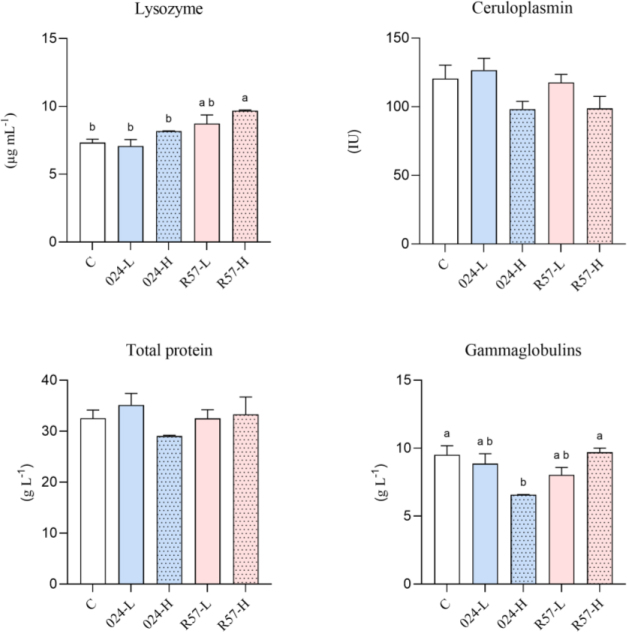
The humoral-mediated immune parameters in serum of rainbow trout challenged with *fliC* gene–repressed (024) and *fliC* gene–expressing (R57) *Yersinia ruckeri* strains. All data are expressed as mean + standard error of the mean. Different letters indicate significant differences (P-value < 0.05), shared or the same letters indicate no significant differences between groups

The activation extents of the innate humoral defence mechanisms in *Y. ruckeri*-infected rainbow trout are compared in Fig. 3. The analysis of the results revealed that lysozyme activity in the serum of rainbow trout was elevated in the R57-H group compared to the control group and both 024 groups. Ceruloplasmin activity showed no significant changes across any of the experimental groups. A similar result was observed in the total protein levels. A lower serum gammaglobulin level was noted in the 024-H group when compared to both the control group and the R57-H group.

### Functional immune cell responses

**Fig. 4.**
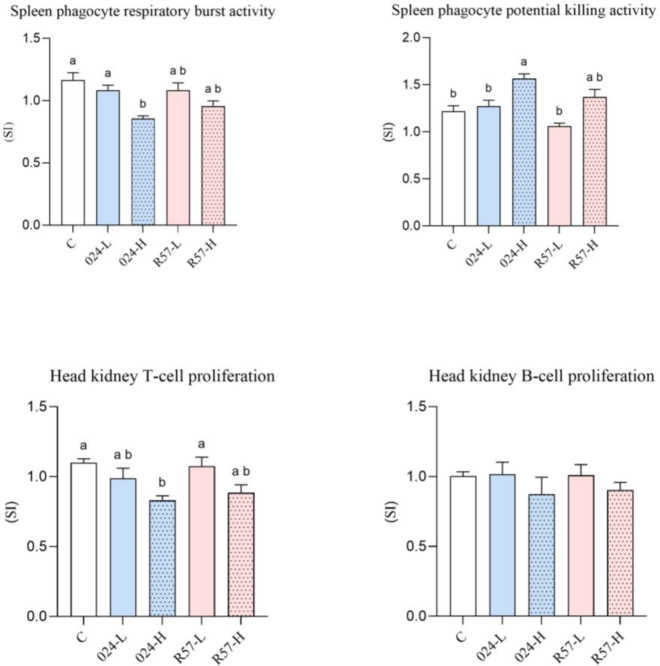
The cell-mediated immune parameters of rainbow trout challenged with *fliC* gene–repressed (024) and *fliC* gene–expressing (R57) *Yersinia ruckeri* strains. All data are expressed as stimulation index + standard error of the mean. Different letters indicate significant differences (P-value < 0.05), and shared or the same letters indicate no significant differences between groups

The activation extents of the innate cellular defence mechanisms in *Y. ruckeri*-infected rainbow trout are presented in Fig. 4. The analysis of the results showed that the RBA of spleen phagocytes was lower in 024-H fish than in the control and 024-L fish. The results of PKA analysis showed stimulation of potential splenic phagocyte killing activity in the 024-H group. The opposite result was obtained for T-cell proliferation, which was suppressed in that group. No statistically significant changes were observed in the analysis of B-cell proliferation.

## Discussion

Bacterial pathogens are microorganisms that possess specialised adaptations, enabling effective interactions with their hosts. They demonstrate the ability to adapt to diverse environmental conditions, resulting in geographic variability and strain diversity. Factors such as environmental changes and selective pressures from ongoing immunisation practices significantly influence the emergence of these strain variations. In host–pathogen interactions, bacterial pathogens employ various mechanisms, including adhesion, invasion, antiphagocytosis and the secretion of proteins or toxins. In Gram-negative bacteria such as *Y. ruckeri* and in Gram-positive species, the flagellum serves as a primary driver of bacterial motility. The flagellar filament, composed of the FliC protein, constitutes the largest structural component of the flagellum. In many cases, the primary function of the flagellum is to locate a suitable site within the host to initiate an infection. The flagellum repeats its primary function to help the bacteria spread the infection to other cells, tissues or even different hosts. Beyond just enabling movement, the flagellum’s motility system has evolved to play crucial roles in specific bacteria. It helps them sense environmental conditions, adhere to target sites, invade host cells and secrete effector molecules. Nevertheless, the flagellum is helpful also in some circumstances by functioning not at all: repression of the flagellar apparatus is frequently observed in motile pathogens and is believed to be a necessary strategy to evade flagellin-mediated recognition by the host immune system (5, 20).

This flagellar repression–mediated immune evasion enhances virulence in *Y. ruckeri*, as epizootic outbreaks select for strains that evade host detection. Such strains, when dominant, drive the high mortality rates observed in acute ERM episodes. Regarding mortality, the impact of *fliC* gene expression was evident in the present study, with the *fliC*-repressed strain inducing higher mortality rates. Consistently, the severity of clinical symptoms and anatomical changes varied according to the strain; however, the size of infectious dose administered also had impact. Notably, the group infected with the strain lacking *fliC* gene expression exhibited more pronounced symptoms and anatomical alterations. These differences were particularly evident in groups receiving the lower infectious dose, as the time required for symptom development was extended. An *Escherichia coli fliC* deletion mutant also displayed enhanced pathogenicity compared to wild-type strains, resulting in an earlier time to death, higher tissue load and severe bacteraemia in infected mice (18). It was also observed that *Salmonella* Typhimurium showed increased virulence after flagella deletion (21), which is consistent with our results.

The relative virulence of the two *Y. ruckeri* strains and the lysozyme production they induced were investigated in this study for possible correlations. The increased production of lysozyme, an antimicrobial enzyme crucial for pathogen elimination, is one of multiple pathways of pathogen recognition. The recognition process is critical for triggering the innate immune response and subsequent host immunity. The level and activity of lysozyme is, therefore, an indicator of innate immunity. This enzyme targets the peptidoglycan layer of bacterial cell walls, leading to bacterial lysis. Lysozyme is found in various biological fluids, including mucus, lymphoid tissues and plasma, and its expression is widespread across different tissues. During bacterial infections, an increase in lysozyme activity is typically observed, as documented in infections caused by *Y. ruckeri*, among other pathogens (23). Our research indicated that in the rainbow trout sera, lysozyme activity was significantly elevated in fish infected with the *Y. ruckeri* strain expressing the *fliC* gene. In contrast, no statistically significant change in lysozyme activity over the control group level was observed in fish infected with the strain lacking the flagellin gene. This confirms that *fliC* expression is important for pathogen recognition and initiation of the immune response.

The role of ceruloplasmin, another innate immune protein, was also investigated for possible correlations with *fliC* expression. Ceruloplasmin is a protein predominantly present in serum, synthesised primarily in the liver of fish. Its structure comprises multiple binding sites for various molecules, including numerous amines, and is suitable for its many roles. Ceruloplasmin functions primarily as a catalyst in redox reactions within plasma and exhibits bactericidal activity. Its ferroxidase activity reduces the availability of free iron, thereby limiting bacterial proliferation. Despite limited data regarding ceruloplasmin activity levels in the context of bacterial infections in fish, existing analyses suggest that *Y. ruckeri* infection induces variable responses in ceruloplasmin concentrations. For example, a significant increase in serum rainbow trout ceruloplasmin levels was reported in one investigation (23), while a decrease was observed in the same species in other studies (10). In our investigation, however, we found no significant changes in ceruloplasmin activity among any of the infected groups when compared to the control group.

We also observed no changes in total protein level among any of the infected groups compared to the control group. Circulating blood proteins play a crucial role in providing essential building materials for the body and perform various vital functions. They help maintain osmotic pressure, regulate pH, transport various metabolites and are involved in the humoral immunity of fish. Total protein content is a straightforward and accessible measure commonly used in health and welfare studies involving fish. However, because of the diverse roles of blood proteins, correlating total protein levels with specific health issues can be challenging. Specific protein fractions may provide more accurate insights into fish biology and health. The primary protein fractions in fish include albumins, alpha-globulins, beta-globulins and gamma-globulins (2). Albumins function as transport proteins and contribute to regulating blood volume by maintaining osmotic pressure in body fluids. The alpha- and beta-globulin fractions are classified as acute-phase proteins, with their levels typically fluctuating in response to inflammation and disease states. Recombinant *Y. ruckeri* flagellin has been shown to upregulate various pro-inflammatory cytokines. However, the responses of acute-phase proteins to flagellin generally peak within the first few hours after stimulation (31). Thus, it is likely that our total protein level results reflect normalisation two weeks post infection. Lastly, the gammaglobulin fraction consists of circulating immunoglobulins, which are primarily involved in the humoral immune response of fish (2). No changes were detected in the expression of immunoglobulins in the spleen of rainbow trout following primary intraperitoneal infection with *Y. ruckeri* (26). Similarly, Soto-Davila *et al*. (28) demonstrated that IgM and IgT levels did not significantly differ between control fish and those infected with *Y. ruckeri* in samples taken from the spleen. Our results also indicated no change in globulin levels in the group infected with the *fliC*-expressing strain. In contrast, we observed a decrease in this parameter after infection with the *fliC*-repressed strain.

Complementary to the examination of serum-mediated immunity, we also investigated the other primary defence mechanism of non-specific immunity in fish, cell-mediated immunity. The activation of phagocytic cells underlies cellular immunity, with neutrophils and macrophages serving as the principal cell types involved in phagocytosis. During this process, stimulation of the cell membrane initiates the production of microbicidal free oxygen radicals or reactive oxygen species (ROS) in a phenomenon known as the respiratory burst. Horne and Barnes (13) described how isolates of *Y. ruckeri* produced superoxide dismutase and catalase, enzymes that enable the bacteria to survive within macrophages by evading phagocytic killing. When interacting with these immune cells, *Yersinia* spp. can impair phagocytosis, inhibit respiratory burst and induce apoptosis (8). Consequently, it is not surprising that our results indicated reduced respiratory activity in the group infected with the *Y. ruckeri* strain which expressed the *fliC* gene. However, this reduction left the activity not significantly different from that of the control group. In contrast, respiratory activity was significantly reduced in the group infected with the *fliC*-repressed strain compared to the control group, which may suggest an increase in cellular infection and apoptosis. Cell death is critical for maintaining tissue homeostasis by eliminating stressed and infected cells, thereby preventing pathogen replication. Our findings indicate that the group infected with the strain expressing no *fliC* gene exhibited enhanced PKA in its splenic phagocytes. This observation is likely attributable to the release by dying cells of elevated levels of ROS into the extracellular space as their membranes degrade (24). This would agree with reports of other authors, for whom the flagellate bacteria showed a higher capacity to inhibit apoptosis. For instance, the flagellin of *Salmonella* inhibited apoptosis of intestinal epithelial cells during intestinal infections as a disease-limiting agent (29), as well as delayed human neutrophil apoptosis (27).

We also examined lymphocyte proliferation to assess adaptive immune responses. Lymphocytes are essential components of the immune response, predominantly located in immune-related tissues and organs. Naive T cells are responsible for mediating cellular immunity, while naive B cells are involved in humoral immunity. Our results indicated no statistically significant changes in B cell proliferation; however, T cell proliferation was suppressed in the group infected with the *fliC*-repressed strain. These two lymphocyte types employ distinct mechanisms to combat pathogens and collaborate to initiate the immune response. Receptors on T cells remain anchored to the cell surface, whereas B cells present their antigen receptors on their membranes and can secrete them as immunoglobulins. In the context of intracellular pathogens, antibodies secreted by B lymphocytes cannot directly neutralise target cells and require assistance from T lymphocytes to lyse infected cells and expose the pathogens. Consequently, the synergy between the cytotoxic activity mediated by T lymphocytes and the production of neutralising antibodies by B lymphocytes is critical for effectively eliminating intracellular infections (34). Research indicates that *Y. ruckeri* is a facultative intracellular pathogen. Similar to many members of the *Enterobacteriaceae* family, it has the capacity to invade non-professional phagocytic cells. It can survive inside macrophages both *in vitro* and *in vivo*, with bacterial numbers continuing to increase post-infection. This intracellular survival strategy confers a significant advantage by shielding the bacterium from immune detection. However, the molecular mechanisms underlying *Y. ruckeri*’s ability to survive in both intracellular and extracellular environments remain poorly understood. In the extracellular space, the structural domain of the flagellin protein is recognised by Toll-like receptor 5 (TLR5) expressed by antigen-presenting cells and T cells. This recognition triggers the expression of several genes that are essential for the host’s defence mechanisms. While TLR5 effectively recognises extracellular flagellin, it is unable to detect flagellin that has penetrated into the host cytosol, where the pathogen becomes inaccessible for activation of B lymphocytes and the humoral immune response (31). Alternatively, upon introduction into the host cytoplasm, flagellin is detected through a different innate immune pathway – the nucleotide-binding and oligomerisation domain–like receptor interleukin-1ß converting enzyme protease activating factor (Ipaf) (33). This means that pathogenic bacteria must either reduce or switch off their flagellar expression or evade the immune system by hiding their flagella in order to survive and thrive in the eukaryotic host’s innate immune system.

Although ERM is one of the most devastating diseases affecting farmed rainbow trout, knowledge regarding the infection dynamics and pathogenicity of its primary cause, *Y. ruckeri*, remains limited and fragmented. Most studies on its virulence are either descriptive or rely on genomic and proteomic approaches based on sequence comparisons with better-characterised pathogenic species. Each new study contributes to a growing body of knowledge aimed at elucidating this pathogen’s characteristics. It is likely that new roles for the bacterial flagellum during pathogenesis will be revealed as our understanding of pathogenic life cycles and processes continues to grow. A more comprehensive understanding of its virulence mechanisms may provide a foundation for developing novel antimicrobial strategies against this economically significant fish pathogen.

## Conclusion

Our study highlights the significant role of *fliC* gene expression in the virulence of *Yersinia ruckeri* and its influence on the immune response of infected rainbow trout. The findings demonstrate that strains lacking *fliC* expression lead to more severe clinical symptoms, increased mortality and a suppressed immune response. These results underscore the dual role of *fliC* in both bacterial pathogenicity and host immune modulation. Understanding the mechanisms by which *Y. ruckeri* evades immune recognition through flagellar repression provides valuable insights for disease prevention and vaccine development in aquaculture. Further research into the interactions between *Y. ruckeri* and the immune system may facilitate novel therapeutic strategies to mitigate enteric redmouth disease and enhance fish health management in commercial aquaculture.

## Supplementary Material

Supplementary Material Details
