# Supplementary material for: Repression of the fliC gene as an immune evasion strategy in Yersinia ruckeri infection of rainbow trout (Oncorhynchus mykiss)
Source: J Vet Res. 2025 Oct 22;69(4):526–34. doi: 10.2478/jvetres-2025-0059 (PMC12767157; doi:10.2478/jvetres-2025-0059)
Supplement: Supplementary file 1 — Supplementary Material Details [file jvetres-2025-0059_sm.pdf]

Supplementary Table S1. Virulence factors identified for *Yersinia ruckeri* strains used to infect rainbow trout and elucidate the role of the bacterial *fli*C gene

**Virulence factor class**	**Virulence factors**	**Related genes**	***Y. ruckeri* 024PP2020**	***Y. ruckeri* R57**
Adherence	Myf/pH6 antigen	*psaC*	+	+
	Type IV pili	*pilN*	+	+
		pilQ	+	+
	Afimbrial adhesin AFA-I (*Escherichia*)	*afaB*	+	+
	Haemorrhagic *E.coli* pilus (HCP) (*Escherichia*)	*ppdD*	+	+
	LPS O-antigen (*Pseudomonas*)		+	+
	Type IV pili biosynthesis (*Pseudomonas*)	*pilU*	+	+
Exoenzyme	YplA	*yplA*	+	+
Invasion	Invasin C	*invC*	+	+
Iron uptake	Yersiniabactin	*irp2*	+	+
	Brucebactin (*Brucella*)	*dhbE*	+	+
	Ent siderophore (*Klebsiella*)		+	+
		entB	+	+
	Enterobactin synthesis (*Shigella*)	*entA*	+	+
	Haeme uptake (*Escherichia*)	*chuA*	+	+
		chuS	+	+
	Mycobactin (*Mycobacterium*)	*mbtH*	+	+
Others	O-antigen	Undetermined	+	+
Protease	Alkaline protease (*Pseudomonas*)	*aprA*	+	+
Secretion system	Flagella (cluster I)	*flgA*	+	+
		flgB	+	+
		flgC	+	+
		flgD	+	+
		flgE	+	+
		flgF	+	+
		flgG	+	+
		flgH	+	+
		flgI	+	+
		flgJ	+	+
		flgK	+	+
		flgL	+	+
		flgM	+	+
		flgN	+	+
		flhA	+	+
		flhB	+	+
		flhC	+	+
		flhD	+	+
		flhE	+	+
		fliA	+	+
		fliC	−	+
		fliD	+	+
		fliE	+	+
		fliF	+	+
		fliG	+	+
		fliH	+	+
		fliI	+	+
		fliJ	+	+
		fliK	+	+
		fliL	+	+
		fliM	+	+
		fliN	+	+
		fliO	+	+
		fliP	+	+

		fliQ	+	+
		fliR	+	+
		fliS	+	+
		fliT	+	+
		fliZ	+	+
		Undetermined	+	+
	T2SS (Yst1)	*yst1C*	+	+
		yst1E	+	+
		yst1F	+	+
		yst1G	+	+
		yst1H	+	+
		yst1I	+	+
		yst1J	+	+
		yst1K	+	+
		yst1L	+	+
		yst1M	+	+
		yst1O	+	+
		yst1S	+	+
	Ysa T3SS	*sycB*	+	+
		ysaC	+	+
		ysaH	+	+
		ysaJ	+	+
		ysaK	+	+
		ysaN	+	+
		ysaR	+	+
		ysaS	+	+
		ysaT	+	+
		ysaU	+	+
		ysaV	+	+
		ysrR	+	+
Toxin	Thermostable haemolysin (TH) (*Aeromonas*)		+	+
Fimbrial adherence determinants	Pef (*Salmonella*)	*pefC*	+	+
		pefD	+	+
	Saf (*Salmonella*)	*safC*	+	+
	Stf (*Salmonella*)	*stfC*	+	+
Glycosylation system	O-linked flagellar glycosylation (*Campylobacter*)	*neuB2*	+	+
Immune evasion	Capsule (*Acinetobacter*)		+	+
	LPS glucosylation (*Shigella*)	*gtrB*	+	+
Stress adaptation	SodCI (*Salmonella*)	*sodCI*	+	+

LPS – lipopolysaccharide; T2SS – type-2 secretion system; T3SS – type-3 secretion system; SodCI – Cu,Zn-cofactored superoxide dismutase isoform I
